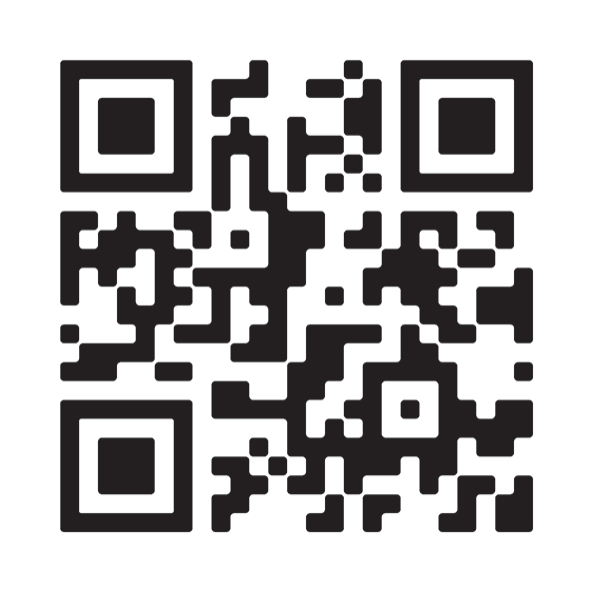# New approach for T-shaped uterus: Metroplasty with resection of lateral fibromuscular tissue using a 15 Fr miniresectoscope. A step-by-step technique

**DOI:** 10.52054/FVVO.13.1.003

**Published:** 2021-03-31

**Authors:** U Catena, R Campo, G Bolomini, MC Moruzzi, V Verdecchia, F Nardelli, I Romito, F Camolo, V La Manna, MM Ianieri, G Scambia, AC Testa

**Affiliations:** Department of Women’s and Children’s Health, Fondazione Policlinico Universitario “A. Gemelli”- IRCCS, Largo Francesco Vito 1, 00168, Rome, Italy; Department of Obstetrics and Gynecology, ZOL Hospitals, Genk, Belgium; Istituto di Ginecologia e Ostetricia, Università Cattolica del Sacro Cuore, Rome, Italy

**Keywords:** Hysteroscopy, T-shaped uterus, metroplasty, mini-resectoscope, dysmorphic uterus, three-dimensional ultrasound

## Abstract

T-shaped uterus is a congenital uterine malformation (CUM), only recently defined by the ESGE ESHRE classification as Class U1a. The uterus is characterised by a narrow uterine cavity due to thickened lateral walls with a correlation 2/3 uterine corpus and 1/3 cervix. Although the significance of this dysmorphic malformation on reproductive performance has been questioned, recent studies reported significant improvement of life birth rates after surgical correction in patients with failed in-vitro fertilisation (IVF) or recurrent miscarriage. The classical surgical technique to treat a T-shaped uterus is by performing a sidewall incision with the micro scissor or bipolar needle, resulting in a triangular cavity.

In this video article, we describe a new surgical technique with a step-by-step method combining three- dimensional ultrasound (3D-US) and hysteroscopic metroplasty in an office setting, using a 15 Fr office resectoscope (Karl Storz, Tuttlingen, Germany), to treat a T-shaped uterus by resecting the lateral fibromuscular tissue of the uterine walls. No complications occurred and the postoperative hysteroscopy showed a triangular and symmetrical uterine cavity without any adhesions.

## Introduction

Female genital malformations are defined as a deviation from normal anatomy due to an impaired development of the Mullerian or paramesonephric ducts. The incidence in the general population is around 0.2-0.4% (Byrne et al., 2000) while in the infertile population is around 4-13% (Grimbizis et al., 2001; Saravelos et al., 2008). Often, uterine malformations are associated with recurrent miscarriage (Grimbizis et al., 2013).

A new classification of uterine malformation was recently published with the collaboration of the European Society of Human Reproduction and Embryology (ESHRE) and the European Society for Gynaecological Endoscopy (ESGE). A new category defined as “dysmorphic uterus” was introduced (Grimbizis et al., 2013). Recently, the Congenital Uterine Malformations by Experts (CUME) group has identified the best measurements that had good diagnostic test accuracy and fair to moderate inter- observer reliability/agreement. The best cut-offs values for these measurements were:

- lateral indentation angle ≤ 130°
- lateral indentation depth ≥ 7 mm
- T-angle ≤ 40°

**Figure 1:**
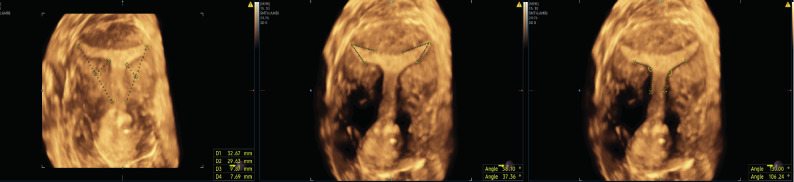
— 3D-US diagnosis of T-shaped uterus, according to CUME consensus (Ludwin et al, 2019): the uterus showed: both lateral indentation depth ≥7 mm in size, bilateral T-angle ≤ 40° and both lateral indentation angle ≤130°. No indentation was detected at fundus level.

They suggest considering as definitely T-shaped uterus when all three criteria are present. (Ludwin et al., 2019) (Figure 1).

Many studies reported poor reproductive outcomes when this malformation was present (Di Spiezio Sardo et al., 2020). In 1992, Golan et al. (1992) demonstrated an association between T-shaped uterus and a high rate of first-trimester miscarriage (47%) and a low rate of full-term delivery (21%).

The management of such patients remain unclear because of the absence of standardised diagnostic criteria and lack of studies comparing surgical treatment with expectant management. Hysteroscopic treatment is often used, however, only few studies have been published evaluating the pregnancy outcome after surgical treatment (Ferro et al., 2018; Di Spiezio Sardo et al., 2020; Alonso Pacheco et al., 2019). Moreover, different surgical techniques have been proposed (Fernandez et al., 2000; Katz et al., 1996; Fernandez et al., 2011; Boza et al., 2019; Di Spiezio Sardo et al., 2016) and most of them included small sample size.

Recently, we have experienced a revolutionary change in our diagnostic and therapeutic approach by introducing the simultaneous use of three- dimensional ultrasound (3D-US) imaging and outpatient hysteroscopy both for the diagnosis and treatment of congenital uterine malformations (CUM). 3D-US imaging has already proved to be the gold standard in the diagnosis of CUM (Bermejo et al., 2010; Salim et al., 2003). Recent technological advances in hysteroscopy allowed gynaecologists to abandon the need for general anaesthesia and cervical dilation, making some procedures such as metroplasty feasible and safe in an “outpatient setting” (Bettocchi et al., 2007; Di Spiezio Sardo et al., 2010). In this new era, 3D-US could be successfully integrated within the endoscopic tower giving rise to the concept of a “digital hysteroscopic clinic” (DHC), where diagnostic and therapeutic moments are united in a one-stop clinic, as recently proposed by Campo (Campo et al., 2018).

In this video presentation, we describe a new surgical technique with a step-by-step method combining 3D-US and hysteroscopic metroplasty in an office setting, using a 15 Fr office resectoscope (Karl Storz, Tuttlingen, Germany), to resect the lateral fibromuscular tissue of the uterine walls, removing the constriction rings in the isthmic area.

## Methods

A 36-year-old woman was referred to our centre following suspicion of a uterine malformation. The patient’s medical history was positive for primary infertility and she underwent three failed in-vitro fertilisation (IVF) attempts. Diagnostic 30° hysteroscopy and 3D-US were performed. Diagnostic hysteroscopy showed a narrow uterine cavity due to thickened lateral walls. Transvaginal 2D ultrasound examination showed a small submucosal uterine myoma (G3) of 10 x 9 x 11 mm in size while both adnexa appeared regular. Using 3D software, the diagnosis of T-shaped uterus was made. According to CUME consensus (Ludwin et al, 2019), the uterus showed both lateral indentation angle ≤130°, both lateral indentation depth ≥7 mm in size and bilateral T-angle ≤ 40°. No indentation was detected at fundus level (Figure 1) .Therefore, due to the fertility desire of the patient and the previous failed IVF attempts, an hysteroscopic metroplasty was proposed. A written informed consent was obtained from the patient before the procedure.

**Figure 2:**
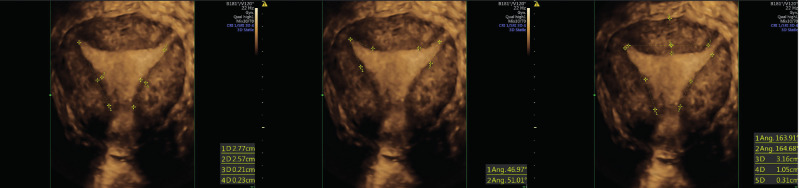
— 3D-US postoperative control showed a regular uterine cavity. According to CUME consensus (Ludwin et al, 2019), the uterus had: both lateral indentation depth <7 mm in size, bilateral T-angle >40° and both lateral indentation angle >130°. No indentation was detected at fundus level.

The patient underwent hysteroscopic metroplasty with a bipolar 15 Fr resectoscope in an office setting under conscious sedation (as achieved with i.v. midazolam 10 millograms and fentanyl 100 micrograms), during the follicular phase immediately after the menstrual phase of the cycle. Metroplasty was performed using a vaginoscopic approach with a bipolar 15 Fr office resectoscope. Saline solution was used as distension medium. Once entered, the uterine cavity showed a tubular morphology. The endometrial mucosa appeared regular. A 90̊ angled bipolar cutting loop was used to resect the lateral fibromuscular tissue of the uterine walls, removing the constriction rings in the isthmic area, first on the right side. During the procedure, the G3 myoma previously described by ultrasound examination was also removed. The incision depth was between 5 and 7 mm and it was determine on the basis of the loop larger diameter (3.5 mm). The incision depth decreased from the cranial to the caudal part of the uterus according to the indentation of the sidewalls determined by 3D-US. The same procedure was repeated on the left uterine wall. No complication occurred. The specimens were sent for histology examination with a diagnosis of endo-myometrium with large fibrotic component. At the end of surgery, the uterine cavity appeared triangular and symmetric. The procedure was ended by controlling any bleeding and by reducing the inflow pressure of the distension medium. At 3D coronal section, uterine cavity appear restored with a normal triangular shape (Figure 2).

After the first deprivation bleeding, postoperative hysteroscopic control showed a triangular and symmetric uterine cavity without any adhesions.

## Discussion

In this article, we describe a new surgical technique with a step-by-step method combining 3D-US and hysteroscopic metroplasty in an office setting, using a 15 Fr office resectoscope (Karl Storz, Tuttlingen, Germany), to resect the lateral fibromuscular tissue of the uterine walls, removing the constriction rings in the isthmic area.

Different surgical techniques have been proposed to treat the T-shaped uterus. Şükür et al. (2018) proposed a lateral metroplasty performed with conventional 26-Fr resectoscope and monopolar needle electrode. Di Spiezio Sardo et al. (2015) proposed an hysteroscopic metroplasty performed with the HOME-DU (Hysteroscopic Outpatient Metroplasty to Expand Dysmorphic Uteri) technique, characterised by lateral and antero- posterior incisions on the constriction rings. This technique was performed using a 5-mm diameter continuous flow hysteroscope with an oval profile and a 30° fore oblique telescope and a 5 Fr bipolar needle to make the incisions. The same authors also suggested that longitudinal lateral incisions may also be performed using 5 Fr scissors to cut the fibromuscular constriction rings. Ferro et al. (2018) described the technique using the 5 French scissors to perform the lateral wall incision.

Regarding the use of the miniresectoscope, it was already proposed to treat isthmocele (Casadio et al., 2019) and uterine septa (Fascilla et al., 2019). Indeed, the small diameter of the sheath allow entery into the uterine cavity without cervical dilatation and so without anatomical distortion of the defect. According to Fascilla et al., (2019) resectoscopic metroplasty was performed with uterine septum excision. In particular, they initially incise the septum using an “L-shape” bipolar electrode up to the fundal area. Then, the septum was longitudinally transected into 2 parts, forming 2 triangles on the anterior and posterior uterine walls, with the base on the fundus and the apex facing the internal cervical os. At the end of the procedure, using a 90° angled bipolar loop, they resected the 2 triangles in parallel, uninterrupted, long strips from the fundus to the apex.

Our surgical technique to treat the T-shaped uterus, combines the benefits of miniaturised instruments and bipolar energy. With the 15 Fr office resectoscope (Karl Storz, Tuttlingen, Germany) we directly entered the uterine cavity without cervical dilatation. We used a 90° angled bipolar cutting loop to completely resect the lateral fibromuscular tissue of the uterine walls, removing the constriction rings in the isthmic area. We believe that resection of the lateral fibromuscular tissue with a mini- resectoscope, instead of making a conventional incision using a bipolar electrode, diode laser or cold scissor, may result in better anatomical restoration of a triangular shaped uterine cavity. Thus, our described technique may be an option to perform a hysteroscopic enlargement metroplasty with the goal of restoring the uterine cavity to its normal size and volume. In our case, the anatomical postoperative outcome was assessed on 3D ultrasonography as satisfactory according to CUME consensus, with a triangular shape cavity clearly apparent after the procedure (Ludwin et al., 2019), (Figure 2).

Whilst our study demonstrates the feasibility of a new approach adding to the existing literature pertaining to hysteroscopic metroplasty, it should be borne in mind that the evidence for reproductive benefit is scant. In the last three years, small, uncontrolled observational studies have been published demonstrating that hysteroscopic metroplasty in infertile patients with a T-shaped uterus may enhance reproductive outcomes (Ferro et al., 2018; Ducellier-Azzola et al., 2018; Di Spiezio Sardo et al, 2020; Alonso Pacheco et al., 2019). However, to date there is a lack of controlled observational or randomised studies evaluating the reproductive outcomes after hysteroscopic surgical treatment compared to expectant management. This uncertainty should be discussed with women with subfertility and a T-shaped uterus considering undergoing the procedure.

## Conclusion

We propose a new surgical approach to treat the T-shaped uterus with a step-by-step method combining 3D-US and hysteroscopic metroplasty in an office setting, using a 15 Fr office resectoscope (Karl Storz, Tuttlingen, Germany). The small diameter of the sheaths makes the surgical procedure faster and easier compared with previous reported surgical procedures. This technique may allow the lateral fibromuscular tissue of the uterine walls to be completely removed while also removing the constriction rings in the isthmic area. It may also reduce the risk of complications, due to the vaginoscopic approach and the absence of cervical dilatation. Moreover, the use of 3D-US can guide the resection of the lateral fibromuscular tissue with a miniaturised bipolar cutting loop. Long term efficacy and safety of this new surgical technique should be confirmed by further studies before it may be offered as a routine treatment for the T-shaped uterus.

## Video scan (read QR)

https://vimeo.com/465700803/4ba8de0b78

**Figure:**